# Emergence of highly prevalent CA-MRSA ST93 as an occupational risk in people working on a pig farm in Australia

**DOI:** 10.1371/journal.pone.0195510

**Published:** 2018-05-02

**Authors:** Shafi Sahibzada, Marta Hernández-Jover, David Jordan, Peter C. Thomson, Jane Heller

**Affiliations:** 1 School of Animal and Veterinary Sciences, Charles Sturt University, Wagga Wagga, NSW, Australia; 2 Graham Centre for Agricultural Innovation, Wagga Wagga, NSW, Australia; 3 New South Wales Department of Primary Industries, Wollongbar, NSW, Australia; 4 School of Life and Environmental Sciences, The University of Sydney, Camden, NSW, Australia; Rockefeller University, UNITED STATES

## Abstract

**Background:**

The occurrence of livestock-associated (LA) MRSA (ST398) in pig herds has emerged as a threat to occupational safety in many parts of the world. Recently, an outbreak of skin lesions due to MRSA occurred in workers at a pig farm in regional Australia and both the humans and pigs were shown to have a high prevalence of carriage of either the human-strain ST93 or porcine strain ST398. This study closely scrutinises this outbreak to determine factors associated with MRSA carriage amongst the workers.

**Methods:**

Information on potential risk factors was collected from employees by means of a questionnaire. The carriage status of MRSA by workers was assessed by nasal swabs processed using standard laboratory techniques with confirmed isolates subjected to sequence typing. Associations between MRSA carriage in workers and their questionnaire responses were investigated using univariable and multivariable logistic regression.

**Results:**

Nasal carriage of MRSA was identified in 60% (31/52) of participants. Workers having contact with pigs had 24 times the odds of MRSA carriage compared to workers with no direct contact (OR 23.6; CI 5.2–172.8). In addition, the probability of MRSA carriage in workers was significantly (*P* < 0.001) associated with the number of hours in contact with pigs and each hour of contact-time per day increased the risk of MRSA carriage by 1.44 times (CI 1.14–1.96). These associations were significant (*P* < 0.001) for both strains, ST398 and ST93, present on this farm. Using a multivariable logistic regression model that incorporated human exposure to five different pig age groups (dry sows, farrowing, weaner, grower, and finisher) as fixed effects, a significant (*P =* 0.027) increased odds of MRSA carriage was found for persons working with farrowing sows compared with those who did not (OR 6.39, CI 1.23–39.36).

**Conclusions:**

This study shows that workers in close contact with pigs on a pig farm where MRSA is present had a higher risk of MRSA carriage as the number of hours of direct contact with pigs increased. Since we have detected a significant association for the human-derived CA-MRSA ST93, similar to the pig-adapted LA-MRSA ST398, we consider ST93 as a potential occupational risk for piggery workers. The risk of MRSA carriage is greatest when working with the farrowing group; therefore, an emphasis is required on personal protective equipment while working in the farrowing house. The study has ramifications for the conduct of surveillance for MRSA in people exposed to pigs.

## 1. Introduction

*Staphylococcus aureus* is a component of the normal flora of humans and some livestock. The organism is a common cause of skin and soft tissue infections, ranging from mild to invasive and life-threatening. *S*. *aureus* strains continuously evolve and adapt to new environments by exchanging resistance and virulence genes [1]. Methicillin resistant forms of *S*. *aureus* (MRSA) rose to prominence in the 1960s, as the cause of nosocomial infections unresponsive to standard therapy and capable of being carried by hospital staff [2]. So called “hospital-acquired MRSA” (HA-MRSA) infections have now evolved to be both highly infectious and resistant to a large number of antibiotic classes [3]. In the 1980s, MRSA were isolated from cases without any recent exposure to health care environments and were termed “community-associated MRSA” (CA-MRSA) [4, 5]. Although CA-MRSA are typically resistant to fewer antibiotic classes than HA-MRSA, they can carry genes encoding for virulence factors resulting in occurrence of severe disease in otherwise healthy people [6]. In 2005, a new MRSA lineage known as “livestock-associated” MRSA (LA-MRSA) was isolated from pig farmers. Since then, the frequency of reports of LA-MRSA carriage has dramatically increased in certain classes of livestock (especially in pigs) and exposed humans. Although transmission to humans, resulting in carriage and infections, has been reported for LA-MRSA [7], it appears to be less likely to cause disease compared to human-adapted strains [8]. LA-MRSA is presently recognised to be comprised of several clonal complexes including CC8, CC9, CC97, CC5 and CC398 [9] with the latter the most frequently isolated of which ST398 is the most common strain type. Europe has been recognised as having a higher ST398 prevalence in people with livestock contact compared to other parts of the world [10]. In Asia ST9 is the predominant LA-MRSA although ST398 is also present [11].

ST398 has been isolated from a variety of animals, and its carriage has been observed in persons in close contact with MRSA infected or carrier animals. However, this strain is most commonly found in pigs and people working on pig farms and is thus also referred as porcine MRSA [9, 12]. The majority of studies have reported that simply having pig contact and living or working on a pig farm are important risk factors for carriage of ST398 [10, 12]. Moreover, the probability of human carriage of porcine MRSA has been shown to increase with increasing frequency of pig contact, stocking density of pigs, and MRSA prevalence in pigs [13, 14]. Recently, a high prevalence of MRSA carriage has also been found in pigs and people working on a pig farm in regional Australia, where two different strains, ST93 and ST398, were identified in people and pigs [15]. The farm had been recognised as it had experienced recurrent clinical MRSA infections among employees over a three-year period and because of an unusual mixture of MRSA clones present. This study investigates the factors associated with MRSA carriage (overall and strain specific) amongst staff members working on the outbreak farm which represents a unique natural experiment for comparing and contrasting the behaviour of human- and pig- adapted forms of the organism in human host.

### Ethics approval

Participation was voluntary, and signed informed consent was obtained from each participant. Approval for the recruitment of human participants into this study was granted by the Charles Sturt University Human Research Ethics Committee (Protocol number 2015/016). Approval for the sample collection in pigs was obtained from the Animal Care and Ethics Committee in Charles Sturt University (Protocol number 14/096).

## 2. Methods and methodology

### 2.1 Data collection

The pig-production enterprise involved in this study was identified as the focus of an MRSA outbreak by the New South Wales Department of Health due to the recurrent detection of MRSA in farm workers affected with clinical staphylococcal disease. A cross-sectional study was commenced in 2015 investigating microbiological and epidemiological aspects of MRSA carriage among piggery employees was conducted in 2015. Recently, we reported a high MRSA carriage amongst workers on this facility with a mixture of sequence types being present and some novel antibiograms [15]. This study, focusing on the epidemiology of the MRSA outbreak, examines the risk factors associated with carriage of MRSA in staff members.

The pig enterprise had two sites (site-A and site-B) geographically separated by approximately 40 km, with a total workforce of 52 employees and with workers moving between both farms. All workers at each site were approached to participate in the study. Participation was voluntary, and informed consent was obtained from all participants. Participation involved collecting a single swab from the external nares and completing a questionnaire.

The questionnaire addressed individual information such as age, sex, level of education, ethnicity, number of years working with pigs, number of months working on the current piggery, main role within the piggery, number of hours working with pigs per week, age group of pigs in contact, and contact with another animal species. The questionnaire also collected self-reported medical history, such as previous and current MRSA infections, hospitalisation, and current or previous antimicrobial treatment. Questions regarding personal hygiene, knowledge and behaviour with respect to antimicrobial use and attitude regarding the potential for MRSA emergence were also asked. The questionnaire was completed individually, with a member of the research team available for clarifications. The questionnaire is available from the corresponding author upon request.

Nasal swabs were collected from all participants on the pig farm followed by isolation of MRSA in the laboratory as described in Sahibzada, Abraham (15). In short, MRSA was isolated using the method recommended by the European Union Reference Laboratory for AMR and screened for various antibiotic resistance attributes following the Clinical and Laboratory Standards Institute protocols [16]. Species identification was performed by matrix-assisted laser desorption / ionisation time-of-flight mass spectrometry (MALDI-TOF MS). Subsequently, all presumptive-MRSA isolates were further confirmed by PCR for identification of *nuc* and *mecA* genes. In addition, DNA microarray, whole genome sequencing, and multi locus sequence typing (MLST) were performed on all MRSA isolates.

### 2.2 Data analysis

Data were entered into Microsoft Excel, and descriptive and statistical analysis were performed with the statistical package R [17] and the package ggplot2 used for graphical display of data [18]. The associations between MRSA carriage and factors were investigated using univariable and multivariable logistic regression models. An alpha level of 0.05 was used as a significance criterion for all statistical tests. The overall model fit was assessed using a likelihood ratio test that derives the *P*-values using a χ^2^ distribution. Odds ratios and confidence intervals for potential risk factors were calculated from the logistic regression models by exponential transformation of the coefficients and its intervals using commands *coef* and *cofint*.

Initially, associations between the different potential risk factors with the presence of MRSA carriage were investigated using univariable logistic regression. The following risk factors were assesed: pig contact (yes/no), intensity of contact (hours per week in direct pig contact), total number of years working with pigs, number of months working on the current study farm, and working on either site-A or site-B (mutually exclusive).

The relationship between MRSA carriage and a participant’s main role on the farm was also assessed. The main role variable had five levels classified as administrative, pastoral, feedmill, maintenance and pig shed workers. An administrative worker was someone dedicated to working in an administration building situated at least 200 m away from the pig sheds. Pastoral workers were those working in the agriculture and cropping section. The feedmill workers only worked in the feedmill. None of administrative, pastoral or feedmill workers reported coming into direct contact with pigs. Maintenance workers were those performing maintenance jobs inside as well as outside the pig sheds, such as fixing fences, water troughs, feed lines and feeders. Some maintenance workers also reported on functioning of the effluent treatment system and helped to move pigs between sheds. Pig shed workers were those who worked directly with pigs in different sheds categorised for different pig age groups. Separate univariable models were also fitted to determine relationship of MRSA in piggery workers that perform a variety of jobs (using each type of job as a separate binomial predictor) including cleaning/washing sheds, moving pigs between sheds and for transportation, feeding, vaccinating, medicating, marking piglets, and assisting farrowing. Pigs were raised in separate sheds based on their produciton stage: dry sow, farrowing, weaner, grower, and finisher. Relationships between MRSA carriage in workers and working with certain pigs’ age groups were also examined considering each age group as separate binomial predictors (yes/no). Relationships between pig contact and carriage of a specific strain (ST93 and ST398) were also assessed using separate univariable analyses. Firstly, the association was determined for one strain (i.e. ST93) by excluding positive records from the counter strain (i.e., ST398) in the baseline model and then comparing the carriage of that strain with MRSA non-carriage. Then the same process was repeated for the second strain.

Participants’ health-related factors were also assessed for association with MRSA carriage which included the history of MRSA diagnosis, hospitalisation in the last twelve months, chronic disease, smoking, consumption of alcohol and personal hygiene. The factors related to the participants’ demography, knowledge and perception toward antibiotic usage and MRSA infection were also included in the analysis.

In addition, two multivariable nested models with predictor variables nested within a single factor ‘*pig-contact’* were used to assess the association with MRSA carriage in people. In one model, working (yes/no) with any of the five pig age groups (used as five separate factors in the model) was assessed, these being nested within ‘pig-contact’ (yes/no). In another model, worker participation in various activities as separate binary variables (yes/no) were assessed, namely feeding, medicating, vaccinating, performing artificial insemination, marking piglets, assisting farrowing, cleaning/washing sheds, treating effluent, and moving pigs between sheds, all these being nested within the ‘pig-contact’ status. For both nested models, all selected variables were entered in the model first, and then a backwards elimination procedure based on *P*-value associated with the Wald statistic was used. The likelihood ratio chi-square test in conjunction with Akaike's information criteria (AIC) was used for checking the actual model fit on each step. In addition, these multivariable models were compared with forward selection, and this resulted in the same variables being selected as in the backward elimination method.

## 3. Results

A total of 52 pig workers (all workers at either site) participated in the study, including 77% male and 23% female workers. The majority identified themselves as Caucasian (61%) followed by Indigenous Australian (22%), and Asian (12%) background. Approximately half of the participants (*n* = 27) were over 40 years of age. In relation to education, 52% of participants had completed tertiary education such as vocational TAFE (Technical and Further Education) or a University degree. Just over 44% had worked in the pig industry for at least five years. The majority (67%) reported working on the current study farm for at least twelve months. All workers but one reported that they worked for at least five days a week and at least seven hours a day on the study farm.

The univariable analyses identified no significant associations with MRSA carriage for gender, age, ethnicity, number of years working with pigs, number of months working on the current study farm, working on either site-A or site-B, smoking, alcohol consumption, history of hospitalization in the last twelve months, chronic disease, and previous MRSA diagnosis (Supporting Information S1 Table). There was also no association detected between MRSA carriage and occupational hygiene practices, individual’s knowledge and perception of MRSA emergence on the farm. The risk factors that have been found significantly associated with MRSA carriage are explained in the following sections.

### 3.1 Work related exposure factors

#### Pig exposure

**Fig 1:**
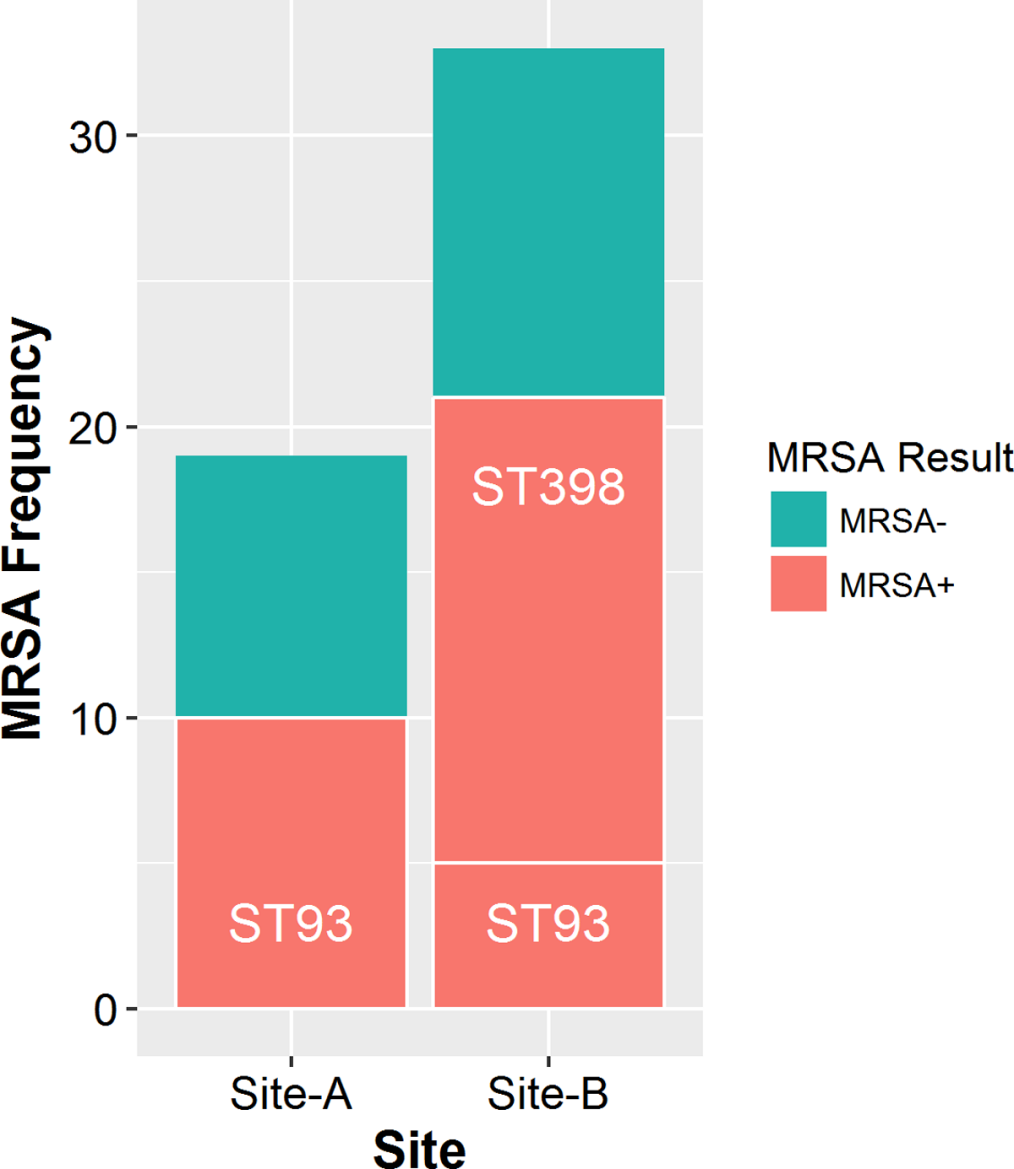
Distribution and frequency of ST93 and ST398 carriage amongst piggery staff on two sites (site-A, site-B) of a single pig enterprise in Australia with a recurrent MRSA outbreak in humans.

Nasal MRSA carriage was identified in 31 of the 52 (60%) participants. The typing results revealed two strain types: ST398 (16.13%) and ST93 (83.87%). On site-A only ST93 was found, whereas both ST93 and ST398 were found on site-B (Fig 1).

**Fig 2:**
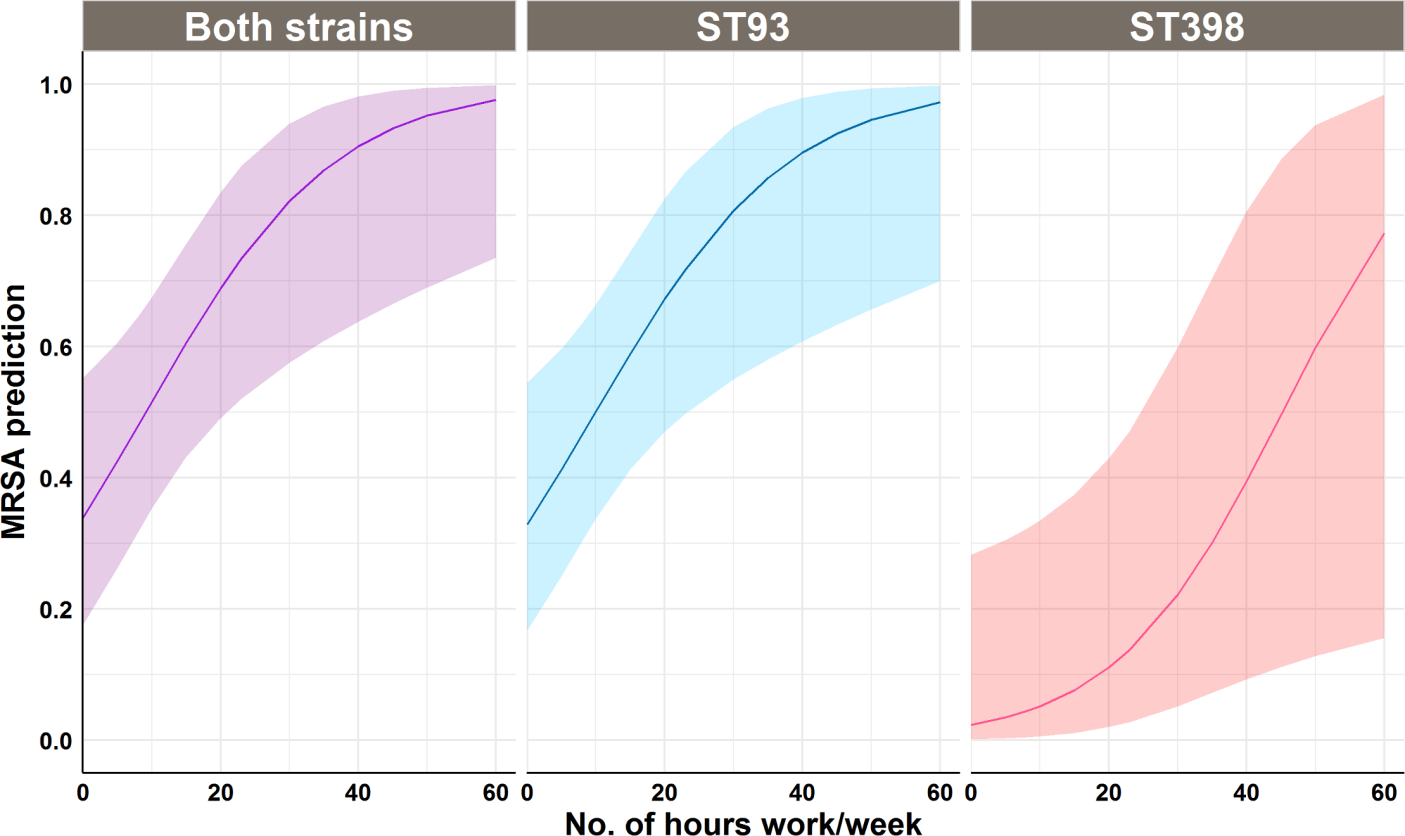
Logistic regression model predictions (and 95% CI) of the probability of MRSA carriage (both strains, ST93, and ST398) in piggery staff for the number of hours per week spent in direct pig contact in a piggery in Australia with a recurrent MRSA outbreak in humans.

Firstly, we investigated if pig exposure was associated with MRSA carriage in people working on the farm. A univariable logistic regression analysis identified a significant (*P<*0.001) association between pig contact and MRSA carriage in farm workers. There was almost 24 times increased odds of MRSA carriage for those working in direct contact with pigs compared to those with no pig contact (OR 23.6; CI 5.2–172.8). Similarly, the number of hours worked with pig exposure was significantly (*P <* 0.001) associated with MRSA carriage in workers with an increasing odds of 1.5 times for each hour increase in a day of direct pig contact (OR 1.44, CI 1.14–1.96). The association between MRSA carriage and contact intensity amongst pig workers is illustrated in Fig 2 (Both strains). The association of pig exposure was also assessed for the carriage of MRSA strains in piggery workers. Using univariable models, an increase in odds for carriage of either strain carriage in pig workers was observed in relation to increase in each hour pig contact in a week as shown in Fig 2 (ST93 and ST398).

#### Role and performing specific activities

**Fig 3:**
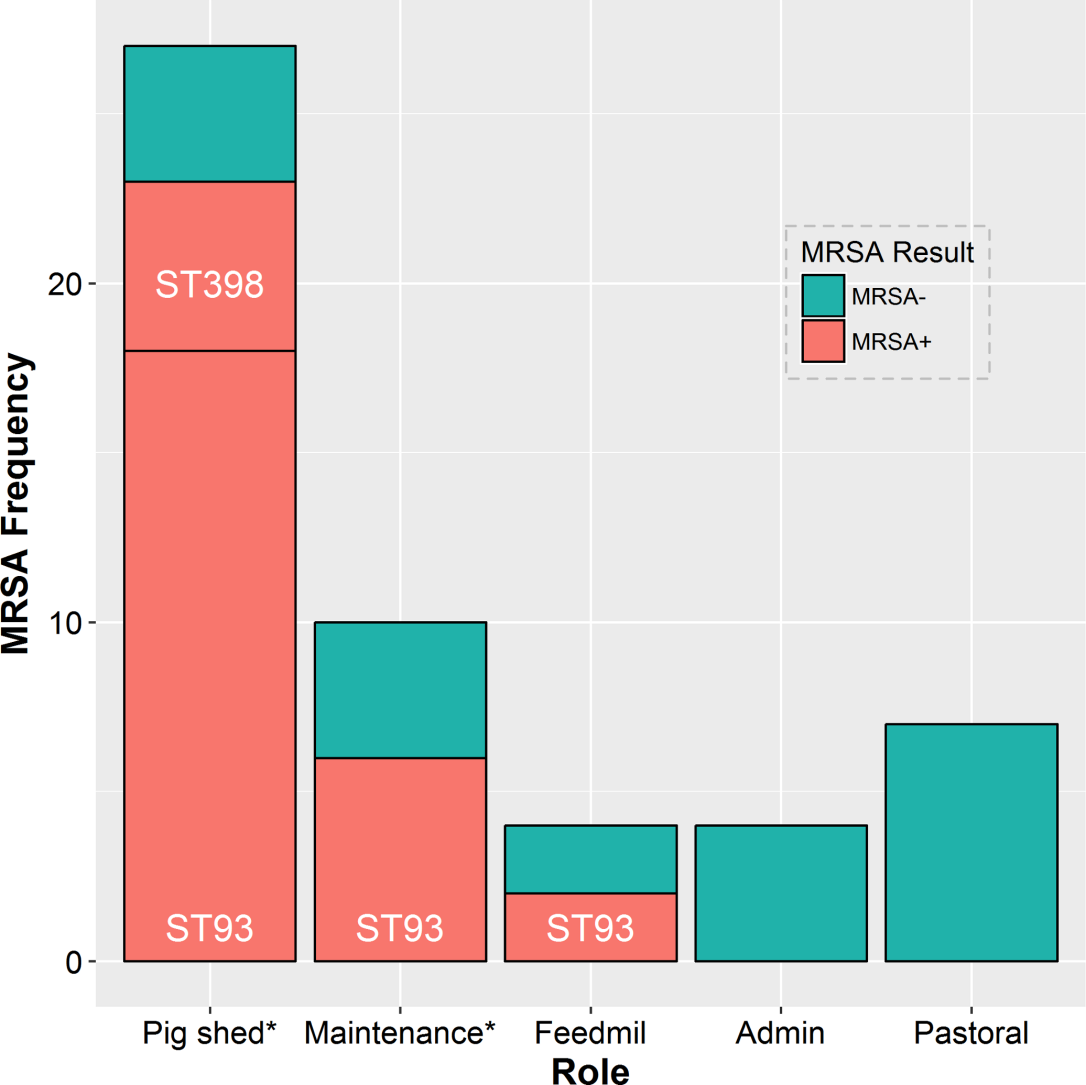
Distribution and frequency of carriage of different strains of MRSA amongst piggery workers performing different roles in a piggery in Australia with a recurrent MRSA outbreak in humans. * Those who come in direct pig contact.

A significant (*P <* 0.001) univariable association was also found between the main role of piggery workers within the piggery and MRSA carriage. While the true effects cannot be accurately estimated for each specific role, due to low numbers of observations in some groups, the prevalence of MRSA carriage was higher (85.2%) for persons working in pig sheds compared to people with other roles (Fig 3).

All workers except people in administrative or pastoral roles performed various activities involving pig contact. We also assessed whether the type of work they were performing had any association with MRSA carriage using univariable models. Except for artificial insemination and effluent treatment, all the activities performed in direct pig contact were significantly (*P* < 0.05) associated with MRSA carriage (details are given in S1 Table). Subsequntly a multivariable model was fitted using all the significant associated activities involved, namely cleaning/washing sheds, moving pigs between sheds and for transportation, feeding, vaccinating, medicating, marking piglets, and assisting farrowing. In the model, the activities were nested within pig contact and assisting farrowing was identified as the only significant (*P* = 0.003) risk factor associated with MRSA carriage in people.

#### Working with a specific pig age group

MRSA carriage in workers was also associated with exposure to a specific age group of pigs. Using univariable analysis a significant association was identified between MRSA carriage and all pig age groups (production pig groups) except for dry sows. The univariable models produced the highest odds ratio for people working with farrowing sows (OR 17.25; CI 4.47–89.2; *P <* 0.001). Significantly higher odds of MRSA carriage were also identified for piggery workers working with finishers (OR 4.94; CI 1.33–24.25; *P <* 0.001), grower (OR 4.53; CI 1.32–18.62; *P =* 0.02), and weaner pigs (OR 4.53; CI 1.32–18.62; *P =* 0.02). A multivariable nested model was used in order to assess the risk posed by each pig age group, when adjusted by the other age groups. After backwards elimination, the model returned only one factor, namely working with farrowing sows, as a significant predictor of MRSA carriage. The model revealed that amongst piggery staff working in pig contact, a person working in the farrowing shed is approximately six times more likely to carry MRSA compared to those not working in the farrowing shed (OR 6.39, CI 1.23–39.36, *P* = 0.027).

### 3.2 Demographic, health, hygiene and perception exposure factors

Using univariable models we also explored the association between MRSA carriage and demographic, health, hygiene practices, and attitudes and perceptions in relation to MRSA emergence and transmission on a pig farm. Level of education was the only demographic factor that was significantly (*P* = 0.02) associated with MRSA carriage. In a multivariable model, after controlling for pig contact, education continued to be significantly associated with MRSA carriage in workers. The estimated odds ratio for MRSA carriage in workers who had high school or lower level of education was 6.36 (CI 1.38–45.94, *P* = 0.02) when compared with those with tertiary education. The proportion of people who had pig contact was almost similar in both education levels, tertiary 49% (*n* = 18) and high school 51% (*n* = 19).

A total of 30% (*n* = 16) of participants reported that they had been diagnosed with MRSA infections on the farm since 2011. In addition, one participant reported being diagnosed in 1993 but was not working on the same farm at the time. The majority of those participants previously diagnosed with MRSA infections on this farm (13/16) were also positive for MRSA carriage in the current study, and most of those (11/13) were carrying ST93. All of those previously diagnosed with MRSA on the study farm reported having had pig contact at the time of diagnosis. A total of 31% of those diagnosed with MRSA on the farm required hospitalisation.

**Fig 4:**
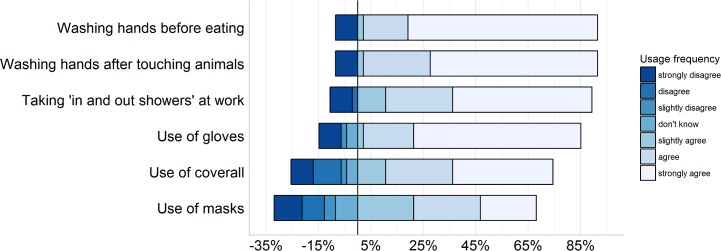
Perception of piggery staff working in a pig enterprise in Australia towards adoption of actions preventing MRSA on a pig farm. The negative percentages (left of the vertical black line) represent percent responses indicating the level of disagreement (‘slightly disagree’ to ‘strongly disagree’, and ‘don’t know’) whereas positive percentages at the right of the vertical black line indicate levels of agreement.

Participants were asked to report the confidence surrounding their personal hygiene and co-workers’ hygiene practices at the work place. Only one third (34%) of participants reported being very confident about their own personal hygiene and their co-workers’ hygiene practices. Most respondents (82%) reported an improvement in their personal or co-workers’ hygiene on the farm after the MRSA outbreak. When participants were asked to rate on a Likert-scale from 0 (minimum) to 5 (maximum) their current level of knowledge about occupational hygiene practices, in terms of reducing the risk of zoonotic transmission of MRSA, the median score was 4. Fig 4 shows that the majority agreed that using proper personal protective equipment like disposable gloves, coveralls and masks could help preventing zoonotic infections. However, some workers (as shown in Fig 4) believed that such practices had no role in preventing zoonotic infections. Less than half (47%) of participants believed that antibiotic use as growth promotion or prophylactic could potentially increase the risk of MRSA emergence on a farm (Figure A in S1 File).

Over two thirds (69%) of respondents showed concern about working on a pig farm and being exposed to zoonoses. Interestingly, a higher proportion (80%) were concerned about their family members getting a zoonotic infection through them than were concerned about acquiring infection themselves. Furthermore, 82% were concerned about their coworkers’ potential exposure to zoonotic diseases. However, when they were asked about the potential for themselves to transmit infections to the pigs, just over half (56%) showed some level of concern, with the rest not being concerned at all (Figure B in S1 File).

In relation to participants’ perception of risky occupational places for MRSA carriage, working in public hospitals and aged care facilities were perceived to pose a very high risk of exposure, followed by working in veterinary clinics and piggeries. Dairy, sheep and beef farms were ranked the least risky places for occupational MRSA carriage in people (Figure C in S1 File).

## 4. Discussion

### 4.1 Pig contact and intensity of contact

Pig exposure was an important determinant for MRSA carriage in this study. We found a 24 fold increase in odds of MRSA carriage in people with direct pig contact, compared to those without direct contact within the study piggery. Direct contact with MRSA-positive pigs has previously been reported as a major risk for MRSA carriage in farmers [14, 19]. In a systematic review that examined 33 studies, animal contact, especially pig contact (OR, 5.91, CI 4.84–7.24), was found to be the most important risk for MRSA carriage in people [10]. Similarly, in a retrospective case control study that assessed 100 patients in each group carrying ST398 and non-ST398 MRSA, pig contact was found to be an important determinant for ST398 carriage (OR 20.46, CI 7.83–64.39, *P <* 0.001), accounting for age, type of infection, hospitalisation, pig contact, and contact with other livestock [20]. However, in contrast to the current study, prior studies have attempted to identify risk factors for MRSA carriage amongst people on a multi-farm level, and carriage under the same environmental condition has rarely been explored.

A temporal association between pig contact and MRSA carriage was found in this study, with the odds of MRSA carriage increasing by 1.44 times for every hour per day increase in direct pig contact. Alejandro, Wietske [21] reported a similar quantitative association between the number of hours worked in pig contact and MRSA carriage in farm workers in a longitudinal study conducted over 18 months on 36 piggeries. On univariable analysis, they reported an odds ratio of 1.82 (1.58–2.06; *P <* 0.001) for each 10 hours per week increase in work. Another study reported an odds ratio of 2.13 (1.65–2.74) for each hour per day increase in pig contact [22], however, it was possible for workers to have other livestock and poultry contact simultaneously with pig contact. Similarly, this study identified a positive association between MRSA strain carriage status and contact intensity (number of hours) for both strains (ST398, ST93).

A significant positive association between hours of pig contact and ST398 carriage has been established [21, 22]. However, such a relationship has never been reported for a non-LA-MRSA. The association found in this study between the CA-MRSA ST93 and contact intensity shows an even stronger and steeper relationship compared to the LA-MRSA ST398 as shown in Fig 2 (ST93 and ST398). ST93 is the most prevalent CA-MRSA in Australia and is frequently isolated from people in community and hospital setups [23–25]. The genetic and antibiotic resistance profile amongst ST93 isolates found on this farm has been described previously [15] and strongly suggests the strain may have become adapted to their new porcine host. Since the likelihood of ST93 carriage found in this study increases as the number of hours in direct pig contact increases, like other porcine MRSA, we classed this strain as an occupational risk, when present in a piggery setting. While ST93 is already a major cause of community-acquired infections in Australia, the adaptation of ST93 to pigs may serve as a reservoir for this strain in isolated cases and may represent a challenge for the pig industry with respect to occupational risk. We found ST398 only in workers who had direct pig contact, which supports findings of other studies that identifies transmission between humans without pig contact seems to be of minor importance for ST398 [26–28].

### 4.2 Working with a specific pig age group

Amongst those working in direct pig contact, MRSA carriage was influenced by exposure to different pig age groups/types of production group. Comparing the odds ratios of working with different age groups produced by univariable models, the highest odds ratio was generated for working with the farrowing group (OR = 17.3). Even in a multivariable model that accounts for working in all five sheds and pig contact, working in the farrowing shed continued to have a strong association with MRSA carriage in piggery workers (OR = 6.4). In another multivariable model that was used to identify the most important risky activities performed by piggery workers, assisting farrowing was recognised as the only significant factor associated with MRSA carriage and this activity is performed only in a farrowing shed. All these analyses indicate that working with farrowing is the most important risk factor when compared to the other age groups. Although the reason for this association is not clear, it could be due to the intense pig contact, since pigs and piglets require intensive and regular handling (birth assistance, tail docking, castration, cutting teeth) and workers are likely to be in much closer contact for longer periods within the working hour with farrowers than with other categories of pigs.

### 4.3 Knowledge and perception

Participants reported that they have mixed knowledge and perception about the risk factors related to the emergence of MRSA on farms. Almost half of the respondents believed that antibiotic use as growth promotants or for prophylactic treatment have no role in the emergence of resistance bacteria. Numerous studies have found a strong correlation between antibiotic usage as growth promotants and the rise of resistance in bacteria on farms [29, 30] that subsequently could transfer to humans.

Overall, attitudes towards control of zoonotic disease were positive, but there were some respondents who were not in agreement that using proper personal protection could help prevent infectious agents transmitting from animals to workers. The workers were intuitively concerned while working with pigs since the majority perceived pig farming as the riskiest occupational place amongst all livestock farming (dairy, beef, sheep) for MRSA exposure and other zoonotic infections. However, the level of concern was comparatively low regarding reverse zoonosis as almost half (44%) showed no concerns about transferring diseases to pigs.

Overall, the knowledge and perception results show a moderate degree of risk awareness and responsibility among participants. However, these responses cannot be directly extrapolated to other commercial piggeries because such response from the participants on this farm might be biased towards knowledge and awareness as, during the course of the outbreak, the majority of them had participated in seminars and training arranged by the government health department. A subsequent survey is required that collects information from commercial piggeries on a larger scale to explore producers’ and workers’ knowledge and perceptions regarding the risk of MRSA emergence and risk management strategies.

## 5. Conclusion

Workers in close contact with pigs had an increased risk of carriage of MRSA, and the likelihood of MRSA carriage increases with the number of hours in direct pig contact. Importantly, these associations have been shown for both the pig-adapted (ST398) and community acquired (ST93) strains of MRSA within this establishment. Therefore, ST93 can be considered as a potential occupational hazard ST93 equal to ST398.

Moreover, exposure of workers to farrowing sows is associated with a higher risk of MRSA carriage, compared with exposure to other types of pigs. The piggery workers are encouraged to use appropriate personal protective equipment and good hygiene practices while working in direct pig contact especially in farrowing house. Greater understanding of the occurrence of human-adapted strains of MRSA in pigs in Australia based on studies of multiple herds is needed.

## Supporting information

S1 Table Outcomes of univariable regression analysis investigating the association of characteristics and practices of piggery workers with carriage of MRSA on a pig farm in Australia with recurrent MRSA infections in humans. (DOCX)

S1 File Figure A. Do you think the following activities are likely to increase the occurrence of MRSA on farm (*abs = antibiotics use) Figure B. As you are working with pigs, how concerned are you that. Figure C. How likely do you think it would be that working at the following workplaces could increase the level of risk of exposure to MRSA? 0 no risk, 5 maximum risk Figure D. Distribution and frequency of MRSA strains found amongst piggery staff working with different pig age groups on two different sites (site-A, site-B) of a piggery in Australia with a recurrent MRSA outbreak in humans. Figure E. Venn diagram showing the overlap of number of piggery workers in different roles in relation to pig age groups and corresponding sheds in a piggery in Australia with a recurrent MRSA outbreak in humans. Figure F. Confidence level of piggery workers about their own and co-workers’ hygiene and protection when working on the farm.
(DOCX)
