# Supplementary material for: Emergence of highly prevalent CA-MRSA ST93 as an occupational risk in people working on a pig farm in Australia
Source: PLoS One. 2018 May 2;13(5):e0195510. doi: 10.1371/journal.pone.0195510 (PMC5931470; doi:10.1371/journal.pone.0195510)
Supplement: S1 Table — (DOCX) [file pone.0195510.s001.docx]

**Emergence of Highly Prevalent CA-MRSA ST93 as an Occupational Risk in People Working on a Pig Farm in Australia**

Shafi Sahibzada^1,2*^, Marta Hernández-Jover^1,2^, David Jordan^3^, Peter C. Thomson^2,4^, and Jane Heller^1,2*^

^1^ School of Animal and Veterinary Sciences, Charles Sturt University, Wagga Wagga, NSW 2678, Australia. ^2^Graham Centre for Agricultural Innovation, Wagga Wagga, NSW 2678, Australia.

^3^New South Wales Department of Primary Industries, Wollongbar, NSW 2478, Australia.

^4^School of Life and Environmental Sciences, The University of Sydney, Camden, NSW 2570, Australia.

* Corresponding authors E-mail: [jheller@csu.edu.au](mailto:jheller@csu.edu.au)

[sshafiullah@csu.edu.au](mailto:sshafiullah@csu.edu.au)

Supporting information S1

S1 Table. Outcomes of univariable regression analysis investigating the association of characteristics and practices of piggery workers with carriage of MRSA on a pig farm in Australia with recurrent MRSA infections in humans.

| **Factors** | **MRSA+ *n* (%)** | **OR (CI)*** | ***P*-Value*** |
| --- | --- | --- | --- |
| **Gender** |  |  | 0.57 |
| Male | 23 (57.5) | **1** |  |
| Female | 8 (66.67) | 1.48 (0.4-6.29) |  |
| **Age (years)** |  |  | 0.53 |
| 20s | 8 (57.1) | **1** |  |
| 30s | 6 (54.5) | 0.9 (0.18-4.52) |  |
| 40s+ | 17 (63) | 1.28 (0.33-4.79) |  |
| **Ethnicity** |  |  | 0.82 |
| Indigenous | 5 (55.56) | **1** |  |
| Asian | 3 (60) | 1.2 (0.13-12.83) |  |
| Caucasian | 18 (66.67) | 1.6 (0.33-7.59) |  |
| **Education** |  |  | **0.02** |
| Tertiary | 12 (44.44) | **1** |  |
| High school | 19 (76) | 3.96 (1.25-13.84) |  |
| **Pig contact** |  |  | **<0.001** |
| no | 2 (13.33) | **1** |  |
| yes | 29 (78.38) | 23.56(5.20-172.75) |  |
| **Perform Cleaning (yes)** |  | 5.82 (1.77-21.97) | **<0.01** |
| **Perform Vaccination (yes)** |  | 8.91 (2.1-62.13) | **<0.01** |
| **Perform medication (yes)** |  | 4.53 (1.32-18.62) | 0.015 |
| **Perform feeding (yes)** |  | 10.39 (2.95-44.59) | **<0.001** |
| **Perform moving pigs (yes)** |  | 13.33 (3.74-56.46) | **<0.001** |
| **Perform artificial insemination (yes)** |  | 2.34 (0.66-9.68) | 0.19 |
| **Perform farrowing assistance (yes)** |  | ∞ | **<0.001** |
| **Perform pig marking/docking (yes)** |  | ∞ | **<0.001** |
| **Perform effluent treatment (yes)** |  | 2.68 (0.77-11.07) | 0.12 |
| **Long-term (working on the study farm) duration of pig contact (years)** |  | 1.02 (0.94-1.14) | 0.62 |
| **Long-term (lifelong) duration of pig contact (years)** |  | 1.06 (0.99-1.14) | 0.07 |
| **Number of hours per week work with dry sows** |  | 1.02 (0.96-1.09) | 0.56 |
| **Number of hours per week work with farrow** |  | 3.25 (1.52-12.67) | **<0.001** |
| **Number of hours per week work with weaner** |  | 1.07 (0.95-1.27) | 0.28 |
| **Number of hours per week work with grower** |  | 1.28 (1.00-1.76) | **0.047** |
| **Number of hours per week work with finisher** |  | 1.86 (1.10-4.62) | **0.01** |
| **Number of hours work per week and** **ST93 carriage** |  | 1.07 (1.02-1.14) | **<0.001** |
| **Number of hours work per week and ST398 carriage** |  | 1.08 (1.02-1.21) | **0.004** |
| **History of MRSA diagnosis** |  |  | 0.08 |
| no | 18 (51.43) | **1** |  |
| yes | 13 (76.47) | 30.7 (0.89-12.61) |  |
| **Chronic disease** |  |  | 0.17 |
| no | 22 (55) | **1** |  |
| yes | 5 (83.33) | 4.09 (0.59-82.18) |  |
| **History of hospitalisation (last 12 months)** |  |  | 0.34 |
| no | 23 (62.16) | **1** |  |
| yes | 3 (42.86) | 0.46 (0.08-2.36) |  |
| **Smoking** |  |  | 0.29 |
| no | 22 (66.67) | **1** |  |
| yes | 7 (50) | 0.5 (0.14-1.8) |  |
| **Drinking alcohol** |  |  | 0.69 |
| no | 6 (66.67) | **1** |  |
| yes | 22 (59.46) | 0.73 (0.14-3.25) |  |
| *****The odds ratio and *P*-values were calculated using univariable logistic regression analysis | | | |
