# Supplementary material for: Emergence of highly prevalent CA-MRSA ST93 as an occupational risk in people working on a pig farm in Australia
Source: PLoS One. 2018 May 2;13(5):e0195510. doi: 10.1371/journal.pone.0195510 (PMC5931470; doi:10.1371/journal.pone.0195510)
Supplement: S1 File — Figure A. Do you think the following activities are likely to increase the occurrence of MRSA on farm (*abs = antibiotics use) Figure B. As you are working with pigs, how concerned are you that. Figure C. How likely do you think it would be that working at the following workplaces could increase the level of risk of exposure to MRSA? 0 no risk, 5 maximum risk Figure D. Distribution and frequency of MRSA strains found amongst piggery staff working with different pig age groups on two different sites (site-A, site-B) of a piggery in Australia with a recurrent MRSA outbreak in humans. Figure E. Venn diagram showing the overlap of number of piggery workers in different roles in relation to pig age groups and corresponding sheds in a piggery in Australia with a recurrent MRSA outbreak in humans. Figure F. Confidence level of piggery workers about their own and co-workers’ hygiene and protection when working on the farm. (DOCX) [file pone.0195510.s002.docx]

**Emergence of Highly Prevalent CA-MRSA ST93 as an Occupational Risk in People Working on a Pig Farm in Australia**

Shafi Sahibzada^1,2*^, Marta Hernández-Jover^1,2^, David Jordan^3^, Peter C. Thomson^2,4^, and Jane Heller^1,2*^

^1^ School of Animal and Veterinary Sciences, Charles Sturt University, Wagga Wagga, NSW 2678, Australia. ^2^Graham Centre for Agricultural Innovation, Wagga Wagga, NSW 2678, Australia.

^3^New South Wales Department of Primary Industries, Wollongbar, NSW 2478, Australia.

^4^School of Life and Environmental Sciences, The University of Sydney, Camden, NSW 2570, Australia.

* Corresponding authors E-mail: [jheller@csu.edu.au](mailto:jheller@csu.edu.au)

[sshafiullah@csu.edu.au](mailto:sshafiullah@csu.edu.au)

Supporting information S1 File


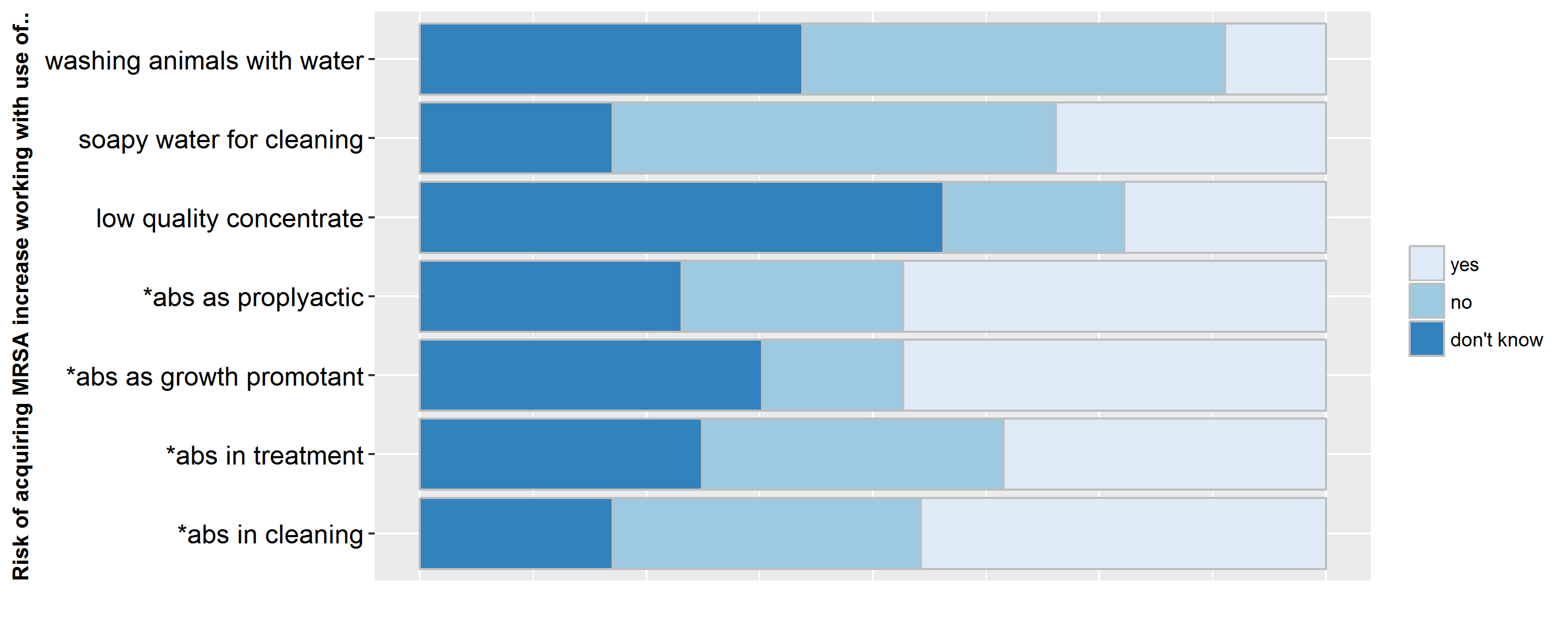


Figure A. Do you think the following activities are likely to increase the occurrence of MRSA on farm (*abs = antibiotics use)


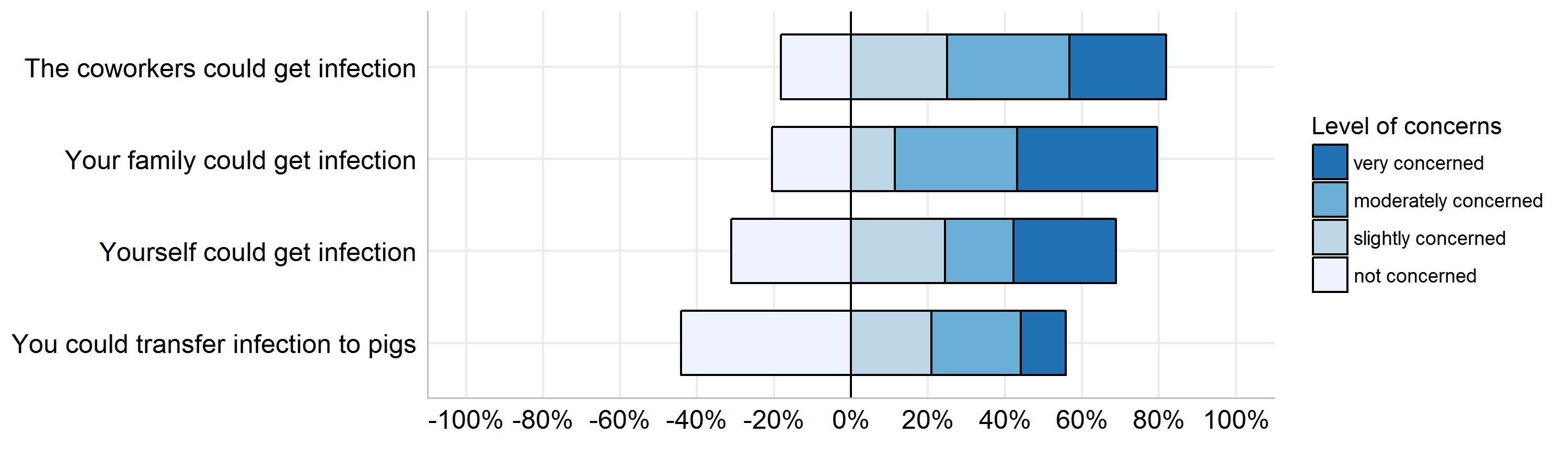


**Figure B.** As you are working with pigs, how concerned are you that …….


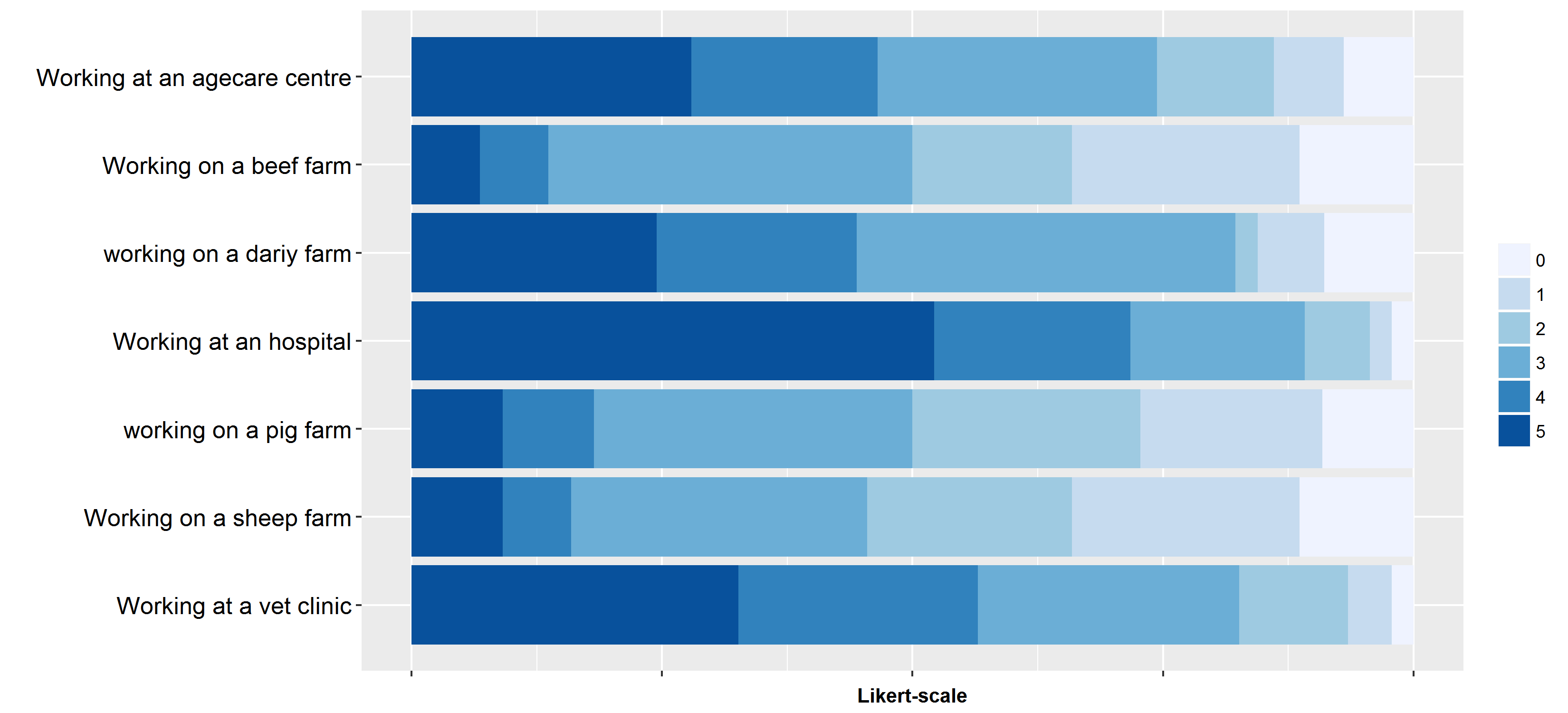


Figure C. How likely do you think it would be that working at the following workplaces could increase the level of risk of exposure to MRSA? 0 no risk, 5 maximum risk


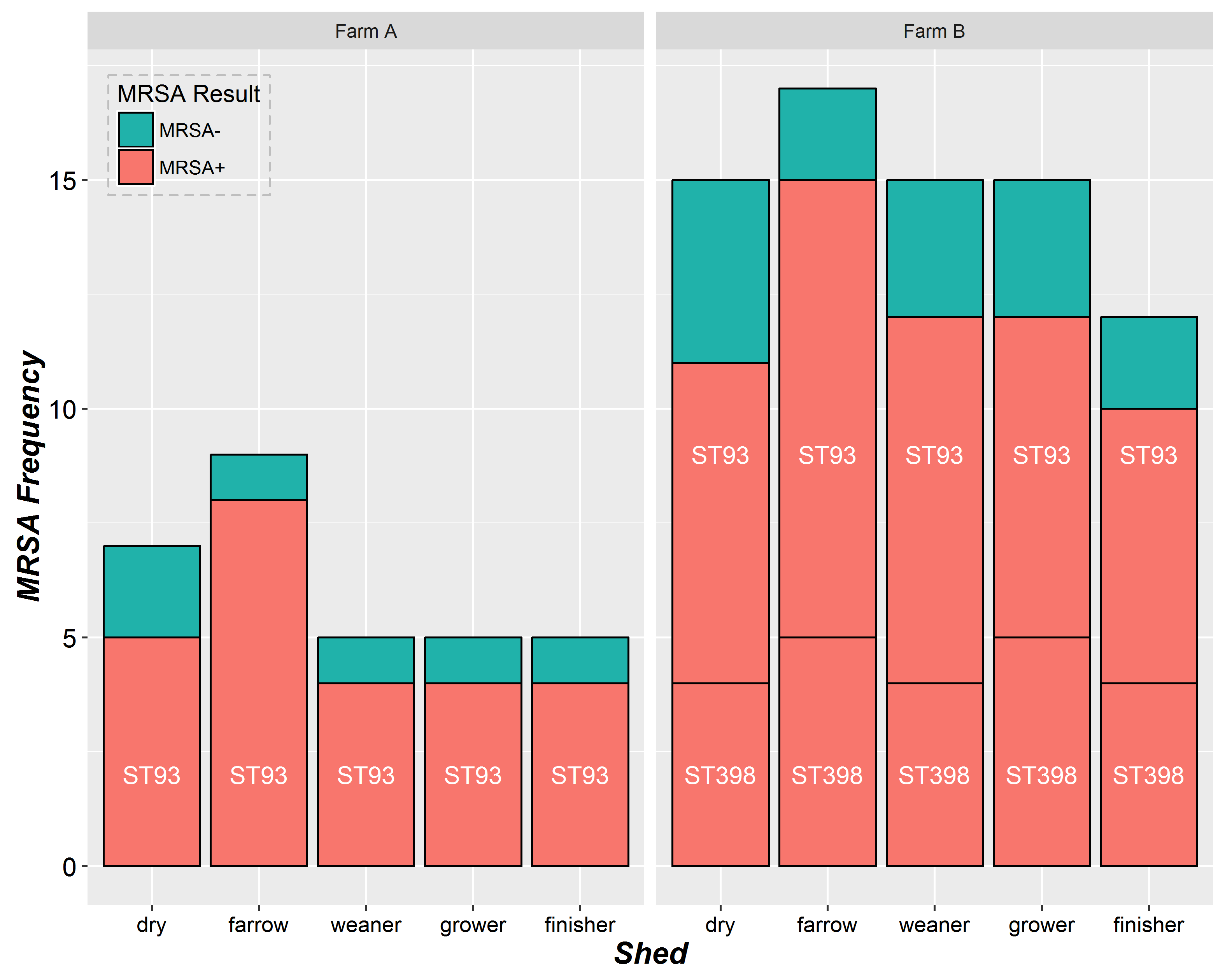


Figure D. Distribution and frequency of MRSA strains found amongst piggery staff working with different pig age groups on two different sites (site-A, site-B) of a piggery in Australia with a recurrent MRSA outbreak in humans.


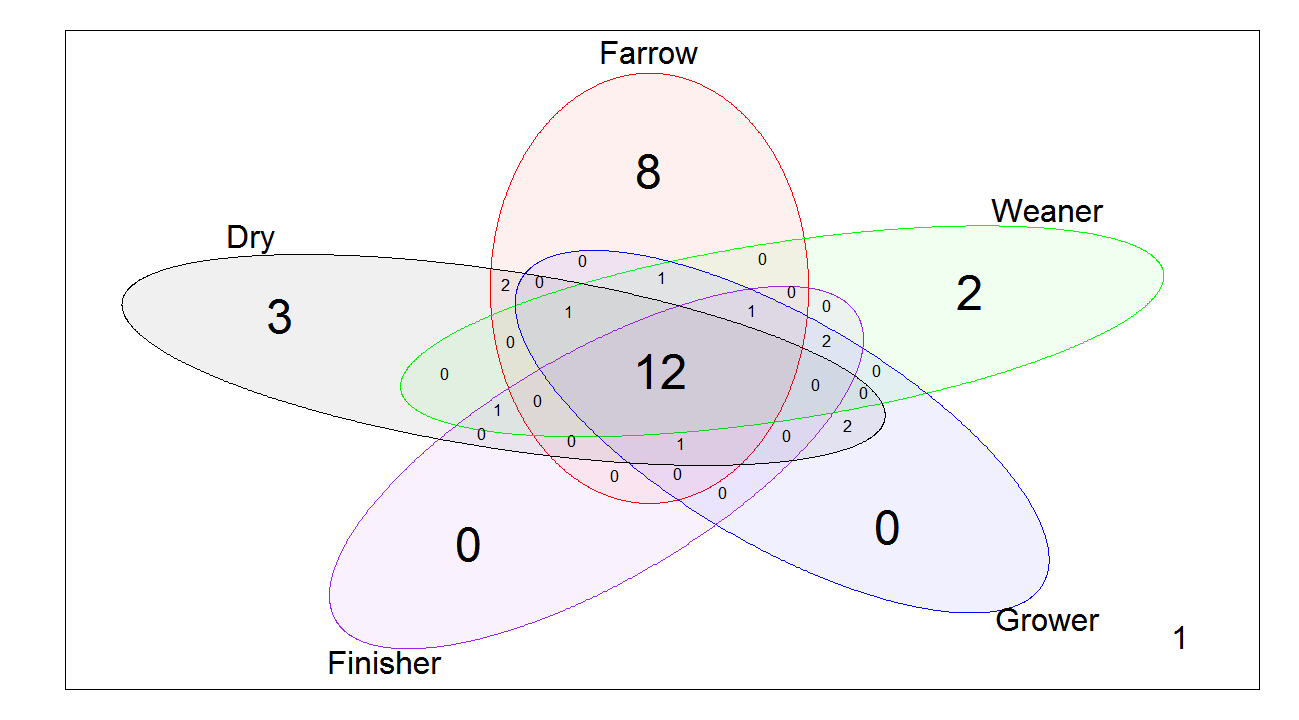


Figure E. Venn diagram showing the overlap of number of piggery workers in different roles in relation to pig age groups and corresponding sheds in a piggery in Australia with a recurrent MRSA outbreak in humans.

**
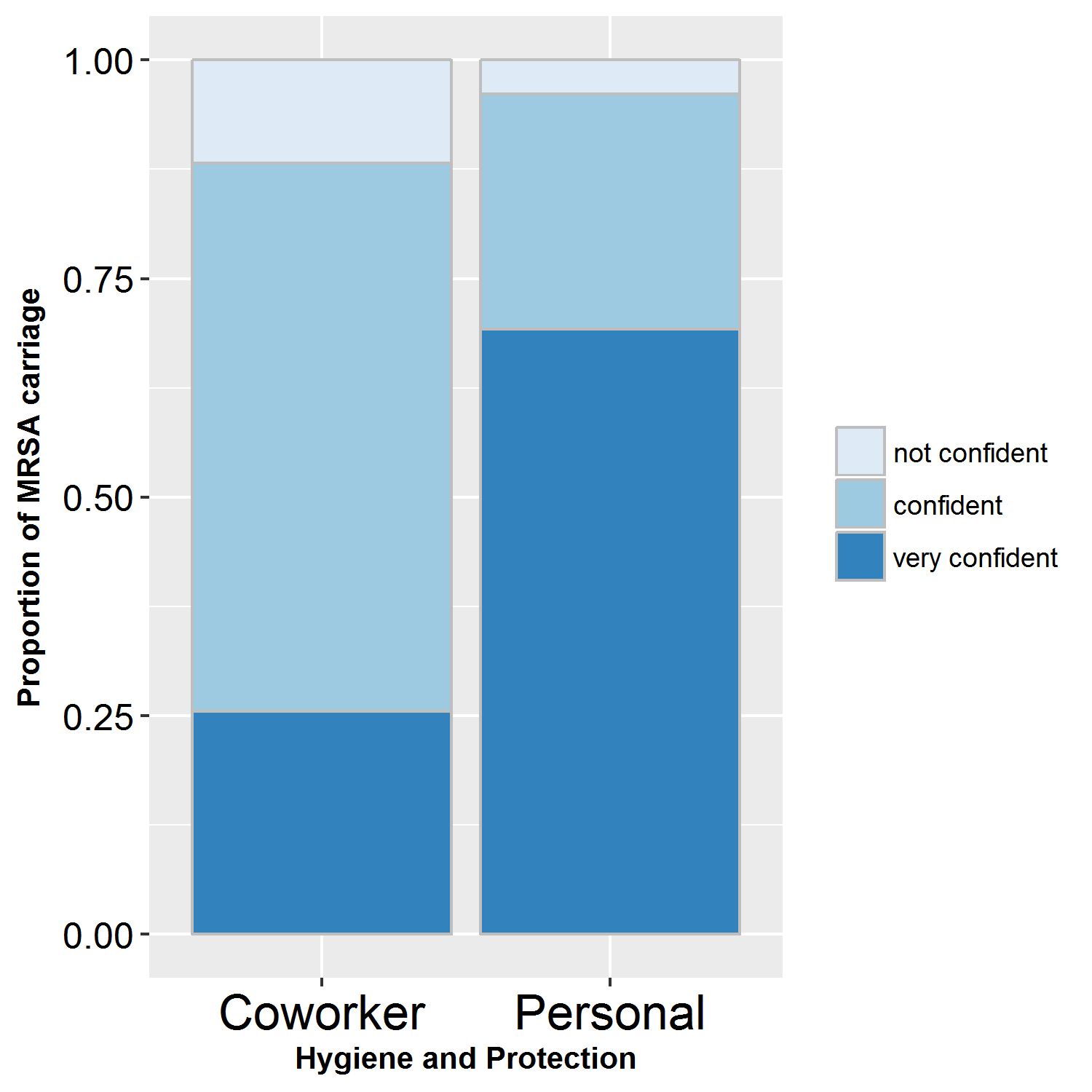
**

Figure F. Confidence level of piggery workers about their own and co-workers’ hygiene and protection when working on the farm.
